# Exploring Impediments Imposed by the Medical Device Regulation EU 2017/745 on Software as a Medical Device

**DOI:** 10.2196/58080

**Published:** 2024-09-05

**Authors:** Liga Svempe

**Affiliations:** 1 Faculty of Social Sciences Riga Stradins University Riga Latvia

**Keywords:** software, artificial intelligence, medical device regulation, rights, digital health

## Abstract

In light of rapid technological advancements, the health care sector is undergoing significant transformation with the continuous emergence of novel digital solutions. Consequently, regulatory frameworks must continuously adapt to ensure their main goal to protect patients. In 2017, the new Medical Device Regulation (EU) 2017/745 (MDR) came into force, bringing more complex requirements for development, launch, and postmarket surveillance. However, the updated regulation considerably impacts the manufacturers, especially small- and medium-sized enterprises, and consequently, the accessibility of medical devices in the European Union market, as many manufacturers decide to either discontinue their products, postpone the launch of new innovative solutions, or leave the European Union market in favor of other regions such as the United States. This could lead to reduced health care quality and slower industry innovation efforts. Effective policy calibration and collaborative efforts are essential to mitigate these effects and promote ongoing advancements in health care technologies in the European Union market. This paper is a narrative review with the objective of exploring hindering factors to software as a medical device development, launch, and marketing brought by the new regulation. It exclusively focuses on the factors that engender obstacles. Related regulations, directives, and proposals were discussed for comparison and further analysis.

## Introduction

Technology has significantly reshaped how we engage with a multitude of products and services, spanning from finance and travel to health care. This progression has introduced a myriad of digital solutions into the market, designed to facilitate the diagnosis, monitoring, and treatment of diverse medical conditions, ultimately enhancing the quality of life of patients. As per MedTech Europe data, there were more than 500,000 medical technologies available in 2023 [1]. Consequently, the role of regulatory measures becomes paramount in safeguarding patient well-being. Nevertheless, regulatory frameworks must adeptly mirror technological advancements to remain pertinent and efficacious, particularly considering the transformative potential of software within the health care domain. Consequently, in the past decades, the novel concept of software as a medical device (SAMD) has been introduced [2].

Historically, Europe has served as the preferred pathway for obtaining medical device approvals. In 2010, Stanford University professor Josh Makower (Makower et al [3]) published survey results of over 200 medical technology companies titled “FDA Impact on US Medical Technology Innovation.” The survey distinctly indicated the predilection for the European Union market over its US counterpart, owing to the expeditious and cost-effective nature of the European Union regulatory process. The European Union regulatory process was noted as significantly more predictable, reasonable, and transparent; 75% of respondents rated their regulatory experience in the European Union as excellent or very good, in stark contrast to the mere 16% who bestowed similar evaluations upon the Food and Drug Administration (FDA). The author aligns with this perspective because Directive 93/42/EEC (hereinafter Medical Device Directive 93/42/EEC [MDD]) had general requirements with fewer regulatory responsibilities.

However, a certain degree of skepticism has arisen concerning the adequacy of the European Union regulation in ensuring patient safety [4]. This concern was addressed by a study conducted in 2016 by Thomas J Hwang (Hwang et al [5]) from Harvard University. The study entailed a cohort analysis, comparing safety issue rates and trial outcome reporting for medical devices approved within the European Union and the United States. The authors concluded that the medical devices approved first in the European Union were associated with an elevated risk and experienced more recalls.

Furthermore, the medical device industry in the past decades has experienced various scandals, casting doubts on the efficacy of the regulatory framework in achieving its overarching goal to ensure patient safety. Foremost among these is the Poly Implant Prothèse (PIP) scandal, an incident that has been prominently cited as a poignant illustration of regulatory deficiencies that can impede the fulfillment of their primary objectives [6].

Also, the MDD, being introduced in 1993, exhibited a notable misalignment with the latest technological advancements and was not sufficient to comprehensively cover SAMD development and launch. The European Union policy makers recognized the shortcomings, namely, outdated regulation, insufficient oversight leading to safety issues, and imprecise requirements, resulting in an “uneven level of protection of the patients, users, and public health,” consequently requiring an improved regulatory framework [7]. Therefore, in 2017, the new Medical Device Regulation (EU) 2017/745 (MDR) came into force, replacing the previous MDD as well as the active implantable medical devices Directive 90/385/EEC (however, the latter is not in the scope of this paper) [8]. As per Recital 1, the new MDR is believed to solve emerging problems as well as to provide transparency and strengthen market surveillance and overall quality of medical devices, benefiting the patient. Undoubtedly, the new regulation clarifies rules for SAMD that were not clearly defined under the MDD. Furthermore, the new regulatory framework applies to all the manufacturers equally, ensuring the same high standards, irrespective of their geographic location. Such standardization and clarification of requirements eliminate disparities that have existed under the MDD, where the interpretation of regulations had varied between different member states, notified bodies (NBs), and manufacturers. However, it is too early to judge if the new regulation has achieved its main goals; as of now, no quantifiable data or research exist to demonstrate if device safety has improved or if the European Union is experiencing fewer product recalls.

## Methods

The paper is a narrative literature review, and it seeks to provide a comprehensive summary of the problems, given the absence of a comparable analysis. A search in Scopus and PubMed databases was performed in November 2022 for articles written in English and published since 2017, the year when the MDR was adopted. The search included two strings: (1) (“medical” AND “device” AND “regulation”) in the title, abstract, and keywords; and (2) (“medical” AND “device” AND “regulation”) in the title. The specific term “software” was intentionally omitted from the search parameters, as its infrequent occurrence in titles, abstracts, or keywords would substantially limit the available pool of literature. Furthermore, it is important to acknowledge that papers can encompass analysis of the MDR in a broader context, yet not explicitly focused on SAMD, and can provide valuable insights, analysis, and conclusions for the research. First, non-English and duplicate articles were excluded, and the remaining articles were evaluated by reviewing their titles and abstracts. Then the articles were fully read, and only papers exploring and analyzing the impact of the MDR were included in the analysis. The initial search returned 341 items in total, out of which 307 items were excluded after screening due to their irrelevance. Thus, 34 papers were deemed applicable for this review.

The paper begins with a concise general overview of the impact of the MDR, exploring market data on innovations and communications from major organizations. It is followed by the main section of the paper (*The Challenges of the MDR*), presenting the analysis of the hindering factors that have been identified in academic literature. These factors were then grouped into thematic dimensions and explored in more detail, including their potential impact on the industry. The *Conclusion* section summarizes the main findings from the literature and discussions in the previous sections and suggests directions for future improvements.

## The Overall Impact of the MDR

The MDR introduces more detailed requirements for all medical devices in terms of development, quality assurance, and clinical evaluation, as well as postmarket surveillance. However, the novel framework has sparked discussions on how these changes impact innovation. A survey conducted by Climedo Health revealed that 81% of the respondents consider the MDR challenging [9]. As per Stern [10], industry regulation often results in delayed or reduced firm entry into markets due to the increased time and costs, eventually reducing incentives to innovate. Thus, while the regulation is introduced to improve patient safety, the slower innovation could paradoxically result in a reduction of patient safety, since the improvements and novel devices might take longer to enter the market. So, the question remains: how to improve regulation without hindering innovation?

The regulatory framework holds particular importance for the pioneer innovators. Manufacturers who develop a first-of-its-kind product experience several disadvantages, such as the lack of specific guidance, the absence of clinical data, and little knowledge that can result in a longer development and approval process as well as additional costs. Stern [10] notes that the first entrant experiences approximately 34% longer regulatory approval process than the first follow-on entrant, also because the regulatory bodies then can release guidance materials that the later entrants can benefit from. This also means increased costs for the first entrants, although they can potentially gain the largest market share.

The industry has already brought considerable attention to the ramifications and challenges associated with the MDR on multiple occasions. In an open letter dated April 15, 2019, the CEO of MedTech Europe, Serge Bernasconi, pointed out the lack of NBs and the unpreparedness of the regulatory system that could result in a shortage of medical devices [11]. The latest appeal was at the end of 2022 when many medical technology CEOs addressed the need for changes or else “Europe faces a scenario where a high number of existing medical devices, upon which patients, hospitals and other health institutions rely, will fail to be recertified on time and therefore risk permanently disappearing from the market. At the same time, the certification of new and improved products is also delayed, resulting in delayed patient access to the benefits of innovation” [12]. The medical associations conglomerate, The Standing Committee of European Doctors, in its letter to the President of the European Commission, Ursula von der Leyen, is being more dramatic, stating that “in some countries, up to 75% of medical devices are at risk of becoming unavailable,” and that the “situation is unacceptable from the point of view of patient safety and quality of care” [13]. The emerging issue is already noticed as the manufacturers exit the European Union market due to various reasons, including increased costs and certification time, which eventually impact the patients as the devices become unavailable [14]. The expression of concern was duly acknowledged and subsequently addressed by Commissioner Stella Kyriakides at the Employment, Social Policy, Health and Consumers Affairs (EPSCO) Council in December 2022, who proposed to extend the transition period [15], and the extension was adopted with Regulation 2023/607, Article 1, by the European Parliament and the Council [16]. But would the extension, which applies only to the existing medical devices, solve all the problems?

Due to the complexity of the MDR, there are various implications for SAMD development. Therefore, the author conducted research exploring currently identified hampering factors. The paper serves as a comprehensive resource for manufacturers to proactively configure their organizational infrastructure and allocate resources in advance. In addition, it is beneficial for health care policy makers in their endeavors to assess and ameliorate industrial policies and practices. This paper will also be beneficial to the government bodies and policy makers who are responsible for the medical technology industry, as it underscores the barriers impeding the evolution of the digital health sector and can contribute to developing tools to foster and maintain innovation.

## The Challenges of the MDR

In the following sections, the hindering factors are grouped into 8 dimensions and are further described and analyzed. Each of the factors has received varying attention in the literature, and their impact on a manufacturer can overlap; however, each one of them can be addressed separately. Table 1 presents an overview of the dimensions and a shortlist of the hindering factors included in each dimension.

**Table 1:** Hindering factors listed and consolidated into dimensions.

Dimensions	Consolidated factors	References
More complex requirements leading to delays	More time is needed for development More financial resources are needed, thus new products will take longer to make available in the market Slowing industry innovation Delays also for recertification	[17-28]
More requirements for clinical evaluation	More manufacturers will need to conduct or repeat clinical trials Harder to prove technical equivalence Most small and medium-sized enterprises lack the financial resources to conduct trials	[17,19,21,22,25,26,29-34]
Increased expenses	Regulatory changes bring additional costs to being compliant Certification and recertification processes are costly Postmarket surveillance seeks more resources	[17-19,24-26,30-41]
Classification issues	Uncertainty if the product is a medical device Classifying correctly Up-classification, which means more complex requirements for development and launch	[17,22,29,32,36,39,42-46]
Limited availability of NBs^a^	The small number of NBs The capacity of existing NBs Interdependence between manufacturers and NBs Poor communication between stakeholders	[21,22,24,26,27,31,33,34,38,39,43,47,48]
Lack of knowledge	Market entry depends on regulatory knowledge Additional costs to acquire competences Lack of knowledge of the European Databank on Medical Devices	[30,31,33,36,37,41]
Lack of guidance	Lack of guidance materials for specific matters Uncertainty about the processes as harmonized standards are not published No provisions for orphan devices	[20,27,28,31,33,40,46,47]
Constraints on software updates	Complicated process to change or add new features Limited possibility of software customization	[17,26,34,36]

^a^NB: notified body.

### More Complex Requirements Leading to Delays

One of the main challenges for all manufacturers is the complex requirements to develop and market a medical device. While the requirements are updated to ensure patient safety, numerous manufacturers may encounter challenges in meeting these requirements. This can lead to several outcomes. For example, first, the new products will take longer to be deployed [17-21], thus slowing the innovation in health care [22-24]. According to MedTech Europe data, the time to certify a medical device under MDR has now doubled to 13-18 months [49]. Second, delays in certification or recertification procedures could potentially result in a reduction of the available product range [18] and the discontinuation of certain products [25,26]. Third, new product launches could potentially be deferred or even canceled, as the emphasis shifts toward the maintenance of existing medical devices [27]. Fourth, some manufacturers might choose to continue supplying their medical devices while opting to withdraw from the European Union market [28].

Given that one of the plausible outcomes is a delayed launch or even the eventual discontinuation of a medical device, there arises a potential jeopardy to the fundamental objective of the MDR, which is to safeguard patient safety [26], since the devices will no longer be available.

### More Requirements for Clinical Evaluation

Another troublesome issue for the manufacturers is the increased need to conduct clinical evaluations [17,19,21,22,29]. Although it derives from the general complexity of the requirements explored in the previous section, this matter is specific and critical for the development thus separated.

For lower-risk medical devices, the manufacturer can provide clinical evaluation without conducting its own clinical trial, yet the device must be proven to be equivalent to the compared device. In those cases, the manufacturer can use other clinical investigations and studies and papers published in peer-reviewed sources. While there are no data available on how many devices are approved based on equivalence in Europe, the data in the United States suggest that it is the majority: 99% of the devices approved between 2015 and 2020 used the 510(k) pathway (the mean number of premarket approvals was 38, compared with a mean of 2982 510(k)s annually) [50].

However, clinical evaluation without conducting own clinical trial is not an option if the developed medical device is innovative, meaning there are no equivalent devices, even if the device in question has low risk. According to the MDR, the equivalence shall be demonstrated in 3 dimensions, namely, technical, clinical, and biological (the latter does not apply to a SAMD).

Technical equivalence means “the device is of similar design; is used under similar conditions of use; has similar specifications and properties including physicochemical properties such as intensity of energy, tensile strength, viscosity, surface characteristics, wavelength, and software algorithms; uses similar deployment methods, where relevant; has similar principles of operation and critical performance requirements” (MDR, Annex XIV Part A, Article 3). The new additional requirement, which is also the most concerning aspect of a SAMD, is to compare software algorithms. While Medical Device Coordination Group 2020-5 [51] suggests the comparison needs to be done only in terms of functionality and clinical performance, not the code itself, algorithms are not public and are the essence (and the unique selling point) of software. Thus, the actual functionality cannot be thoroughly compared, especially if it is an artificial intelligence (AI) and machine learning (ML)–based solution. In the meantime, for implantable and higher-risk devices (Class III), the equivalence can be claimed only if the manufacturer has a contract in place that allows full access to the technical documentation of the equivalent device on an ongoing basis (MDR Article 61(5)). This requirement is almost impossible to fulfill in a competitive market [27].

According to Kearney and McDermott’s [31] research about challenges with clinical evaluation, manufacturers also frequently have issues related to obtaining and understanding the level of clinical data required by the MDR, being reluctant to the more stringent requirements. However, the most common challenge here is the lack of skills and knowledge for preparing the clinical evaluation, which has led to an increase in outsourcing the knowledge (and consequently, increasing the costs).

An apparent consequence of the increased need for clinical trials is increased research and development costs [22,30,32], which also complicates the development process [33] and increases the maintenance costs [34]. In addition, it is no secret that most small- and medium-sized enterprises (SMEs) lack the financial resources to conduct large clinical trials [26].

Consequently, manufacturers are already deciding to exit the European Union market or at least postpone the launch of innovative products [31].

### Increased Expenses

While most of the identified implications result in increased expenses, it is important to single it out and explore its impact on the development and launch of SAMDs. Also, increased costs were highlighted in the Climedo Health survey [9], where 70% of the respondents named it as their greatest challenge, 44% of respondents projected that the MDR will incur additional costs of 5% of their annual turnover, while the same number of respondents plan cost increase by 1% to 5% of their annual turnover.

The regulatory improvements will clearly bring more development process costs [17,31] as well as conformity assessment, certification, and recertification costs [25,26,32-38]. It will also require changes in organizational structure and a need to acquire new competencies, including bringing more talent on board [18,24,30,32,33,39] and seeking additional funding, which is challenging, especially for SMEs.

The new regulatory updates also establish more requirements for the postmarket surveillance process, which brings more costs [32,40]. In addition, the new unique device identification (UDI) system requires adaptation of the existing information systems, also resulting in cost increase [41].

The increased expenses, if not covered by additional investments, can eventually lead to price increases, which means decreased availability of medical devices [17,19,32,39], or in worst-case scenarios it can lead to an exit from the European Union market [32,35]. Some SMEs might need to seek other exit solutions, such as merging with large companies to keep the products running [17,32,33], resulting in less competition and few dominant companies in the market.

### Classification Issues

Before specifying the device class, the manufacturer must define if the developed product is a medical device: software with a medical purpose acting to benefit individual patients. Then the next step is to choose the right class according to the risk level.

**Figure 1:**
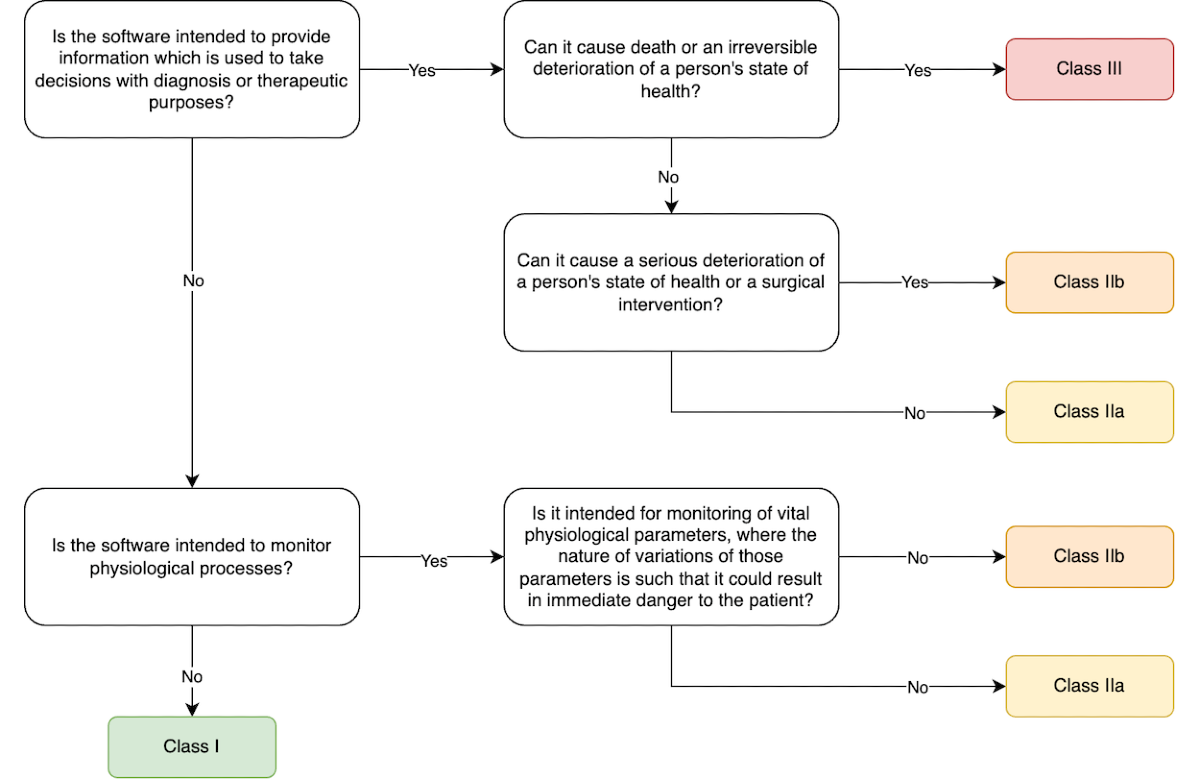
Software as a medical device classification flow.

In comparison to the previous directive (MDD), the MDR separates a rule for SAMDs (Rule 11, Annex VIII, Chapter III, 6.3.; Figure 1).

Yet the classification process has its struggles, such as (1) uncertainty if the developed product is a medical device at all [32,36], thus if it falls within the scope of MDR; (2) choosing the correct class [32,36,42,43]; and (3) for most SAMDs, the new MDR means up-classification [29,32,39,44,45].

The first 2 issues can be addressed with a rigorous regulation study and consultations. This is a one-time issue not impacting the manufacturers in the long term.

However, considering the complexity of the regulatory requirements, SMEs might opt to position their products as health and wellness devices. This strategic approach could allow them to avoid rigorous regulatory scrutiny and lessen the challenges to launch and market their products [46]. In this context, the manufacturer’s envisaged intended use of the device plays the role, rather than its actual use. This perspective is supported by the Court of Justice of the European Union with its decision on Brain Products, wherein it was established that if a product is not intended to be used for medical purposes, Conformité Européenne certification is not required [52]. Yet, the intentional falling out of the regulatory scope means the uncertain quality of the digital health tool, as well as it potentially carrying safety risks to human health, which was the initial cause of the regulatory changes. This concern is important, particularly in light of the increasing reliance of individuals on digital health technologies [46].

The third factor, up-classification, can become troublesome, as most SAMDs will be classified in at least the IIa risk class, and software in the lowest risk class I will remain as an exception [45]. This up-classification entails more complex requirements and certification processes and will result in more time needed for development and subsequent delays in obtaining market access [17,22]. Some manufacturers might choose to eliminate or limit some of the device features to keep the device in a lower-risk class [32], and this might in turn limit the availability of innovative solutions.

The complex requirements as an impediment are explored earlier in this paper.

### Limited Availability of NBs

An NB is an organization that is authorized to perform conformity assessments in accordance with the MDR. Annex VII sets out general, organizational, resource, and other requirements to be met by the NBs, thus the process of establishing an NB can be lengthy and resource-intensive as well.

Consequently, the limited availability of NBs is one of the hindering factors identified, and it has two dimensions: (1) the absolute number of NBs [22,23,25,28,34,39,43,47,48] and (2) the capacity of existing NBs [33,38,43].

As of May 2024, according to the European Commission database, there are 46 authorized NBs to perform assessments following the MDR [53]. Germany and Italy have the highest concentration of NBs, with each country hosting 10. The Netherlands follows with 4 NBs. Finland, Czechia, Turkey, Poland, France, and Sweden each host 2 NBs. Meanwhile, Belgium, Croatia, Hungary, Ireland, Norway, Slovakia, Slovenia, Spain, Cyprus, and Denmark each have 1 NB.

Although the MDR entered into force in 2017, seven years have not been enough to establish a sufficient number of bodies. It is worth pointing out that not all European countries host an NB. For example, Switzerland is the second largest medical technology employer in Europe per capita, or fifth largest in absolute number of people employed [1]; however, it does not host a single NB. Ireland, which has the largest number of people directly employed in the medical technology industry per 10,000 inhabitants, hosts 1 NB. Similarly, France, which is fourth in Europe with the highest direct employment in terms of absolute number of people employed, hosts only 2 NBs. While the international environment does not limit the applicants based on location, and manufacturers can freely choose an NB in any other country, Peter et al [34] suggested that the lack of NBs and poor communication between the manufacturers and NBs can lead to lost market opportunities. However, the authors do not mention any particular or potential examples.

The capacity of existing bodies is also being explored. Because of the low number of NBs and the increased need to acquire a certification, this can result in delays and shortages of medical devices or some manufacturers even leaving the European Union market [48]. The increased time for the certification process has been already mentioned in this paper earlier: according to MedTech Europe data, the time to certify a medical device under MDR has now doubled to 13-18 months. As per the preliminary results of the NBs survey led by Gesundheit Österreich GmbH (Austrian National Public Health Institute) with Areté and Civic Consulting [54], in March 2023, in 86% of cases, the time to acquire the certificates was more than 13 months.

Another factor identified is the interdependence of the NBs with the manufacturers [26], as the NBs would want to ensure their turnover and eventually profit, thus prioritizing the clients who would bring the largest revenue. This argument is especially troublesome for SMEs, as large companies might have the leverage to be certified first, thus leading to delays for SMEs [39]. As per the MedTech Europe report conducted in April 2022, at least 15% and up to 30% of SMEs have no access to an MDR-designated NB [49]. In addition, poor communication between the NBs and manufacturers can lead to potential setbacks during the certification process [31].

Interestingly, Fink and Akra [55] delve into an aspect concerning the regulatory frameworks in the European Union compared with the United States. While this does not pertain specifically to the Regulation, it remains a noteworthy consideration in a broader context. The distinction lies in the centralized approval process in the United States, where the FDA singularly holds authority, and the decentralized process in the European Union, involving multiple NBs. The authors mention the potential risk of different interpretations of requirements stemming from the decentralized nature of the European Union regulatory system.

### Lack of Knowledge

Stern [10] in her paper titled “Innovation under Regulatory Uncertainty: Evidence from Medical Technology” separates 2 factors: technological uncertainty and uncertainty about application content and format. Thus, it is worth exploring both factors separately. (1) Technological uncertainty means the lack of knowledge and understanding of how the innovative product in question works and how it is used in the human body, as well as how the regulator will understand the mechanisms behind it; and (2) the uncertainty about application content and format is related to a lack of guidance for the product assessment phase (including the evaluation of clinical trials and the information needed to submit), and this factor is explored in the next section.

This paper revealed that the lack of knowledge can be an obstacle to entering the market, especially for start-ups [33], and it can be associated with legal risks as well [36]. There is a need to acquire more competencies [31,37], which results in additional costs [30]. Manufacturers need to improve their knowledge and skills to perform postmarket surveillance [33], and better understand the European Databank on Medical Devices system [41].

### Lack of Guidance

Chatterji [56] finds evidence that nontechnical knowledge, such as understanding regulation and marketing knowledge, is of greater importance than technical knowledge. Thus, this impediment should draw the attention of the manufacturers and stakeholders.

The MDR consists of 123 articles and 17 annexes on 175 pages. In comparison, the MDD had 23 articles and 12 annexes on 60 pages. While this suggests that the new regulation shall bring clear requirements and explanations, the actual situation is on the contrary. The Climedo Health survey shows that 59% of the respondents name the lack of clarity as one of their greatest challenges [9]. The unclarity is evident in clinical evaluation [28,31], postmarket surveillance processes [46] and activities [33], or the lack of guidance in general [27,40]. Gilbert et al [20] also specify that there is a need for smarter regulation, particularly for the highest-risk (III) class devices.

Melvin [47] draws attention to the fact that the MDR has no provisions for orphan devices that are intended for rare life-threatening or chronically debilitating conditions. Since the market for these devices is small, it may become economically unfeasible for manufacturers to continue supplying them, potentially leading to their exit from the European Union market.

A significant shortcoming of the MDR is the lack of detailed requirements and guidance for AI solutions, which can become a barrier to their clinical adoption [20]. AI solutions experience rapid growth, including in health care, and the lack of regulation can lead to uncertainty for development as well as compliance. While there is the AI Act that also covers the health care industry, the manufacturers today do not have guidance, which leaves room for their interpretation.

The importance of regulatory guidance and its impact on medical devices is proved by Stern [10], showing an average decrease in regulatory approval times of 2.8 to 6.6 months when comparing innovative firstcomers with their followers.

### Constraints on Software Updates

Opposite to a common hardware medical device, software can be updated regularly. It can vary from a minor update, such as a new data field or color change, to a significant update, such as a brand new feature, delivering a new type of content, or improving the AI algorithm.

This is a considerable difference if we explore the maintenance of a medical device throughout its lifecycle. The MDR now requires an NB’s involvement, namely, if a manufacturer has “any plan for substantial changes to the quality management system, or the device-range covered” (Annex IX, Chapter I, 2.4), or if technical documentation is being changed and “such changes could affect the safety and performance of the device or the conditions prescribed for use of the device” (Annex IX, Chapter II, 4.10). Thus, this requirement to involve the NB if the manufacturer plans updates in the device is considered an impediment [17,26,34], which brings constraints on changing the software or adding new features, as well as limits software customization [36].

Nevertheless, the MDR does not specify what changes should be communicated with the NB, which eventually allows the manufacturer’s interpretation to some degree. However, the Medical Device Coordination Group has provided a guidance document on significant changes regarding the transitional provision under Article 120 [57] to better understand which changes are considered “significant.” It gives the explanation that minor software changes would be adding a new language or fixing bugs. Yet, changes in algorithms shall be considered as a major change, thus requiring the involvement of the NB, and this is burdensome for all AI solutions as the algorithms can change regularly. Furthermore, adding a new therapeutic feature (even if it is only enriching the content base) is considered a significant update, requiring the involvement of the NB.

## Conclusions

This paper is scoped to the impact of the new MDR on the development and launch of SAMDs and does not explore its effect on patient safety. Future research should seek to investigate the benefits and impact of the MDR on increasing patient safety and if the regulatory changes have had the desired effect.

The identified hindering factors of the MDR were consolidated into eight dimensions: (1) more complex requirements leading to delays, (2) more requirements for clinical trials, (3) an increase in expenses, (4) classification issues, (5) limited availability of NBs, (6) lack of knowledge, (7) lack of guidance, and (8) constraints on software updates. Each of the factors has received varying attention, yet any single one can have a critical impact on a manufacturer, thus each company shall evaluate its strengths and weaknesses to sufficiently prepare for development.

The results show that the new regulation heavily impacts the European Union medical device industry, which can lead to either price increases or shortage of medical devices, as well as stifling innovation, which can eventually even harm the patients. Some manufacturers might evaluate the costs and potential revenue and decide to discontinue the devices. Some small start-ups may find themselves compelled to shut down their operations, while other enterprises might opt to exit the European Union market or engage in mergers with more sizable corporations. This trend is supported by Kearney and McDermott’s [31] research showing the first signs of manufacturers either leaving the European Union market or seeking approval for devices in the United States first.

The fact that Europe has lost its appeal is supported by the Boston Consulting Group and University of California, Los Angeles Biodesign report [58], which shows that 89% of surveyed companies consider prioritizing the United States over the European Union. While the registration of digital technologies is quite uncertain for both United States and European Union markets, still 32% of the respondents considered the US pathway rather predictable, which is more than double that of the European Union pathway (15%) [58]. The market changes are alarming for the European Union; thus, the problems must be addressed at the European Union level. Policy makers should reconsider if all the current regulation requirements bring actual value and ensure patient safety rather than build unnecessary burdens to launch innovative digital solutions. Although the transition period for the existing medical devices has been extended, it gives time for the existing medical devices to fulfill the requirements, yet it does not address the issues with complexity, and hence further actions must be taken.

In the meantime, the growing development and approbation of digital health tools, including AI solutions, currently require a more targeted regulatory framework as we see the lack of guidance and knowledge in the domain. The MDR and respective guidelines exhibit limitations in addressing the complexities inherent to most pioneering technologies, thereby AI and ML–based solution manufacturers have room for interpretation of the applicable regulation. To address the regulatory gaps for AI and ML–driven solutions, in March 2024, the European Commission passed the AI Act [59], which will now lessen the legal uncertainties. This legislation covers various domains, and also applies to health care and medical AI, thereby ensuring more robust regulatory oversight within this landscape. Henceforth, manufacturers of AI and ML–driven solutions will be required to ensure compliance with both the MDR and the AI Act. Although the adoption of the act is commendable, in reality, it has introduced new compatibility challenges. For instance, there is uncertainty surrounding the process of providing clinical evidence for certification under the MDR. It appears that AI medical devices will be required to have Conformité Européenne certification before undergoing testing, potentially creating an infinite loop of unmet requirements, or forcing the manufacturers to conduct trials outside the European Union. Therefore, this seeks further discussions and implementation guidelines from the European Commission to help the manufacturers in their compliance journey.

Last but not the least, each European Union member state that aspires to foster the advancement of the digital health sector is encouraged to consider both monetary and nonmonetary assistance to SMEs. Such support mechanisms hold the potential to facilitate a seamless introduction of cutting-edge innovations to the marketplace. While financial assistance might be subject to budgetary constraints, nonfinancial support can be equally pronounced. This encompasses diverse facets, such as the establishment of digital health hubs to facilitate the exchange of knowledge and experience, endeavors aimed at attracting skilled personnel, and the active promotion of educational initiatives.

## Abbreviations

AI: artificial intelligence
EPSCO: Employment, Social Policy, Health and Consumers Affairs
FDA: Food and Drug Administration
MDD: Medical Device Directive 93/42/EEC
MDR: Medical Device Regulation (EU) 2017/745
ML: machine learning
NB: notified body
PIP: Poly Implant Prothèse
SAMD: software as a medical device
SME: small- and medium-sized enterprise
UDI: unique device identification
